# Effectiveness of supervised machine learning models for electrical fault detection in solar PV systems

**DOI:** 10.1038/s41598-025-18802-4

**Published:** 2025-10-07

**Authors:** Ved Khandeparkar, Shreshtha, Senthil Kumar Ramu

**Affiliations:** School of Electrical Engineering, Vellore Institute of Technology, Chennai, Tamil Nadu 600127 India

**Keywords:** PV, Machine learning, Fault detection, Classification, Energy science and technology, Engineering

## Abstract

Even though Photovoltaic (PV) systems have emerged as a viable substitute for non-renewable energy sources, their widespread integration into the electrical grid presents several issues today. On the other hand, various faults are a key concern affecting PV plants’ production and longevity. The current study uses Machine Learning (ML) algorithms such as Decision Tree (DT), Naïve Bayes (NB), Random Forest (RF), Support Vector Machine (SVM) and XGBoost to detect and classify PV errors corresponding to Short Circuits (SC), Open Circuits (OC), Ground Faults (GF), and Mismatch Faults (MF). Simulations were conducted in MATLAB/Simulink to analyse voltage, current, and power variations during fault conditions and study their impact. The proposed results show that the effectiveness of ML in electrical fault detection, with the following classification accuracies: SVM – 97.40%, DT– 97.20%, RF – 97.20%, NB – 97.60%, and XGBoost – 98.0%. The effectiveness of the classification is confirmed through confusion matrices and correlation heatmaps. This research highlights the need for integrating intelligent monitoring, real-time IoT-based detection, and prediction analytics to improve PV system reliability.

## Introduction

Recent reports show that there has been a rise in equipment-related outages in the global solar industry. It’s now 3.13% in 2022 and 4.47% in 2023. This caused a loss in annual revenue of about $4.6 billion, or $4,696 per megawatt (MW). The large-scale projects over 100 MW faced even more difficulties, and the costs increased to $5,000 per MW^1^. The decline in the global solar segment’s overall efficiency of $10 billion of asset underperformance was observable in 2024, indicating a 15% increase over the year. Only the United States faced an over-200% loss in profitability in the last five years, with $5,720 being the average loss per MW per year. These studies show the hardships suffered by investors, project developers, and asset managers and stress the urgency of better maintenance and monitoring systems. If such problems are not solved, the solar power sector’s profitability will inevitably be reduced, and its future growth capabilities will be undermined^2^.

The rapid adoption of PV systems worldwide has resulted in the need for vigorous fault detection and classification methods to guarantee the system’s reliability and long-term operation, which are critically important^3^. The various problems in PV systems, such as OC faults, SC faults, MF, and GF, can produce less power than is supposed to, cause system inefficiencies, and place citizens’ lives in danger. Traditional fault detection technologies can only monitor the hardware, which is time-consuming, expensive, and faults. It is not possible to be completely safe under varying environmental conditions. In this paper, the authors proposed a solution incorporating ML based on simulation-based fault analysis to detect better and classify different faults in PV systems.

*SDG 7: Affordable and clean energy* The proposed work is due to the efficiency and reliability of solar panels by providing ML-based fault detection. By accurately detecting and classifying errors, the system minimizes energy losses, improves performance, and enables continuous solar power generation^4^. It is accomplished by making PV technology more sustainable, efficient, and resilient, which contributes to reaching the goal of universal access to clean and affordable energy^5^.

*SDG 13: Climate action* Optimizing PV systems with advanced fault analysis is the key to climate action, as it reduces emissions. Fault detection is used to detect faults efficiently, and it is the cause of energy wastage by preventing the seamless transition to renewable energies and promoting sustainable solar power generation, which opens the way for sustainability in solar power generation^6^. By the way, it works; the designed system helps to minimize the overuse of solar energy. In conjunction with the reduced emission of greenhouse gases, it helps to battle global climate change and promote a low-carbon energy future^7^.

Due to their improved ability to handle the extraction and classification of feature issues, several AI techniques, such as ML and DL, have been integrated as the fundamental approach of PV defect detection and diagnosis in recent years. For PV system diagnostics and defect detection, several ML approaches have been developed^8]– [9^. Thakfan et al. proposed an AI-based fault detection method for PV systems, highlighting the need for AI-driven solutions to improve classification accuracy^10^. According to the authors, while each AI model, such as neural networks(NN), SVMs, RF, DT, logistical regression, KNN, and naive Bayes, offers distinct advantages for defect identification, no single model can adequately handle all the difficulties PV systems face. Logistic regression and NB are better suited for small-scale systems, while NN and RF models can perform exceptionally well with massive, complicated datasets. Haque et al. introduced a fault diagnosis system using thermography and NN for real-time monitoring^11^. The shortcomings of different condition monitoring and fault diagnostic systems were examined based on different output characteristics and performance factors of PV modules. Signal processing methods based on wavelet transforms were used to extract and identify features in the frequency and time domains. NN methods were used to train the obtained characteristics for categorization.

Building upon these studies, the proposed system employs a supervised learning approach using an RF classifier to analyse key electrical parameters such as current, voltage, and power. The proposed work ensures high accuracy and robustness in fault classification by training the model on real-world fault conditions, improving PV system performance^12^. Each ML algorithm was selected based on how well it handled categorization challenges. DT provides interpretability and simplicity. NB works well with tiny, noisy datasets as it assumes feature independence^4^. RF uses ensemble learning to enhance generalization. When it comes to high-dimensional, non-linear classification jobs, SVM are excellent. Because of its scalability, resilience, and capacity to manage unbalanced data through gradient boosting, XGBoost was included. To provide thorough benchmarking for dependable PV fault classification across various operating settings, these models were selected to assess performance trade-offs regarding accuracy, computational speed, and flexibility.

In^4^, it is addressed how to use machine learning techniques to identify which panels in a 9 × 9 PV array are healthy and which are not. The study does not validate multiple fault categories, including standard, OC, SC, MF, and GF. Most research works concentrate on traditional methods such as artificial neural networks (ANN), linear regression, SVM, fuzzy logic, and defect identification based on thermal imaging. Despite their excellent accuracy, these approaches frequently have limited real-time application and considerable computing cost. The novelty of the proposed work lies in the comparative analysis of five different machine learning algorithms, such as SVM, DT, RF, NB, and XGBoost, for the detection of healthy and four different failure situations. This method has a strong emphasis on classification performance as well as suitability for deployment in solar PV monitoring scenarios with limited resources. The proposed system uses a classifier to detect and classify faults based on key electrical parameters like voltage, current, and power, ensuring high accuracy and robust fault detection.

The proposed system utilizes MATLAB/Simulink-based simulations to analyze systematically different fault types in a 9 × 9 PV array. These simulations provide insights into the behaviour of PV systems under various fault conditions, enabling the visualization of fault impacts through heatmaps. Visualization techniques aid in precise fault localization and predictive maintenance, making fault detection more intuitive and actionable^13^. This approach bridges the gap between theoretical fault modeling and practical implementation, improving the reliability of PV systems^14^.

To promote interpretability and reliability, the rigorous evaluation of the presented work is performed by utilizing essential performance metrics, including confusion matrices, learning curves, accuracy vs. training set size plots, and correlation heatmaps. Hyperparameter tuning, consisting of DT depth parameter tuning and sample size optimization, is also performed to optimize classification performance, reduce false positives, and increase real-world use. The focus on premature fault detection aids decision-making for predictive maintenance strategies that can prevent downtime and catastrophic system failure^15^.

The proposed system presents a cost-effective and scalable solution to traditional fault detection methods, solving industry issues of PV system underperformance, inefficiencies, and revenue losses^16^. As solar energy continues to gain adoption, the results of this research greatly enhance PV system fault diagnosis and facilitate the smooth integration of solar power into contemporary energy grids. Through ML-based classification, MATLAB-based fault simulation, and analysis of real-world data, the work proposed herein presents an innovative, low-cost, and large-scale methodology for improving the sustainability and reliability of solar PV technology.

**Fig. 1:**
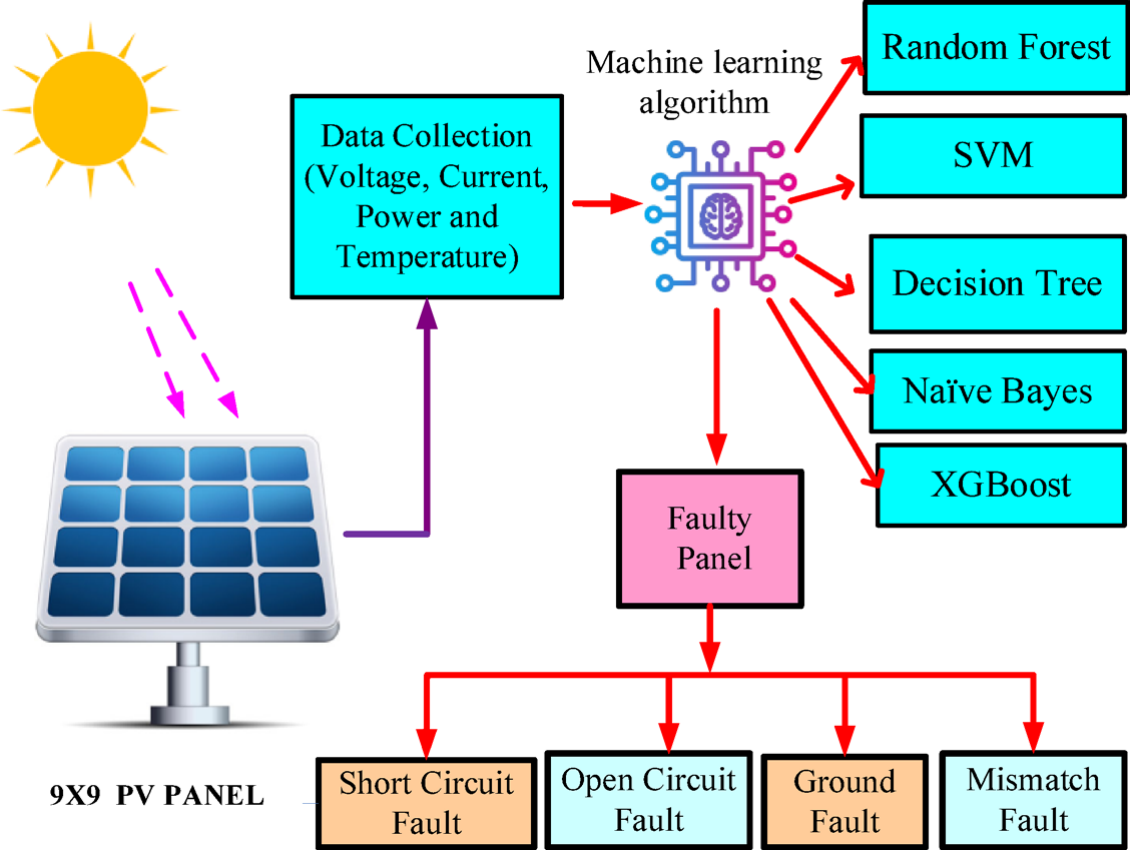
Proposed block diagram for the system.

Figure 1 represents the block diagram of the proposed ML-based PV fault classification system. The figure starts from a 9 × 9 PV panel, which is exposed to sunlight to generate electricity. Parameters like voltage, current, irradiance, and temperature are sensed by the solar panel system, which acts as input data for processing.

The data gathered is input into an ML-based model that examines the performance of solar panels. Various classification methods, such as RF, SVM, DT, and NB, are employed to discern patterns from the data and trigger alarms for anomalies. The ML models assist in making smart predictions about faults in the system. After the ML algorithm processes the data, a fault classifier classifies identified faults into four categories: SC fault, OC fault, GF, and MF. Fault detection aids in diagnosing the PV system, enhances efficiency, and operates the solar power setup. The proposed work integrates advanced ML with simulation-based fault analysis.

The proposed system uses MATLAB/Simulink-based simulations to analyze various fault types, including OC, SC, GF and MF, within a 9 × 9 PV array. Fault maps and heatmaps improve fault localization and predictive maintenance, making fault detection more intuitive and actionable.

To ensure reliability, the proposed work undergoes rigorous evaluation using confusion matrices, learning curves, and correlation heatmaps. Early fault detection is the main feature that makes a PV system go for maintenance; this, in turn, decreases downtime and increases the life of the PV system. By combining ML, MATLAB simulations, and real-world data analysis, this work sets new benchmarks for sustainability and reliability in solar PV technology.

The contributions of this work can be outlined as follows:

- Simulations were conducted in MATLAB/Simulink for the PV array to analyse the impacts of various faults based on voltage, current, power outputs and temperature.
- Supervised algorithms such as DT, RF, SVM, NB, and XGBoost to examine the ML-based fault detection classification.
- Correlation heatmaps and fault maps were developed to improve predictive maintenance by providing visualization and exact localization of defects in PV arrays.
- PV panel faults are classified using the proposed ML technique compared with conventional methods.

The remainder of the article is structured as follows: Sect. “Mathematical modelling of PV modules” covers the mathematical model of the PV system. Sections “Fault analysis in PV system” and “Machine learning-based fault detection in PV systems” describe the types of faults used for this work and the proposed ML Algorithms, respectively. Section Result and discussion deals with the findings of the study. Compared with existing works, the proposed work is presented in Sect. “Discussion”. Finally, Sect. “Conclusion” deals with the conclusion of the article.

## Mathematical modelling of PV modules

### Solar cell modeling techniques

**Fig. 2:**
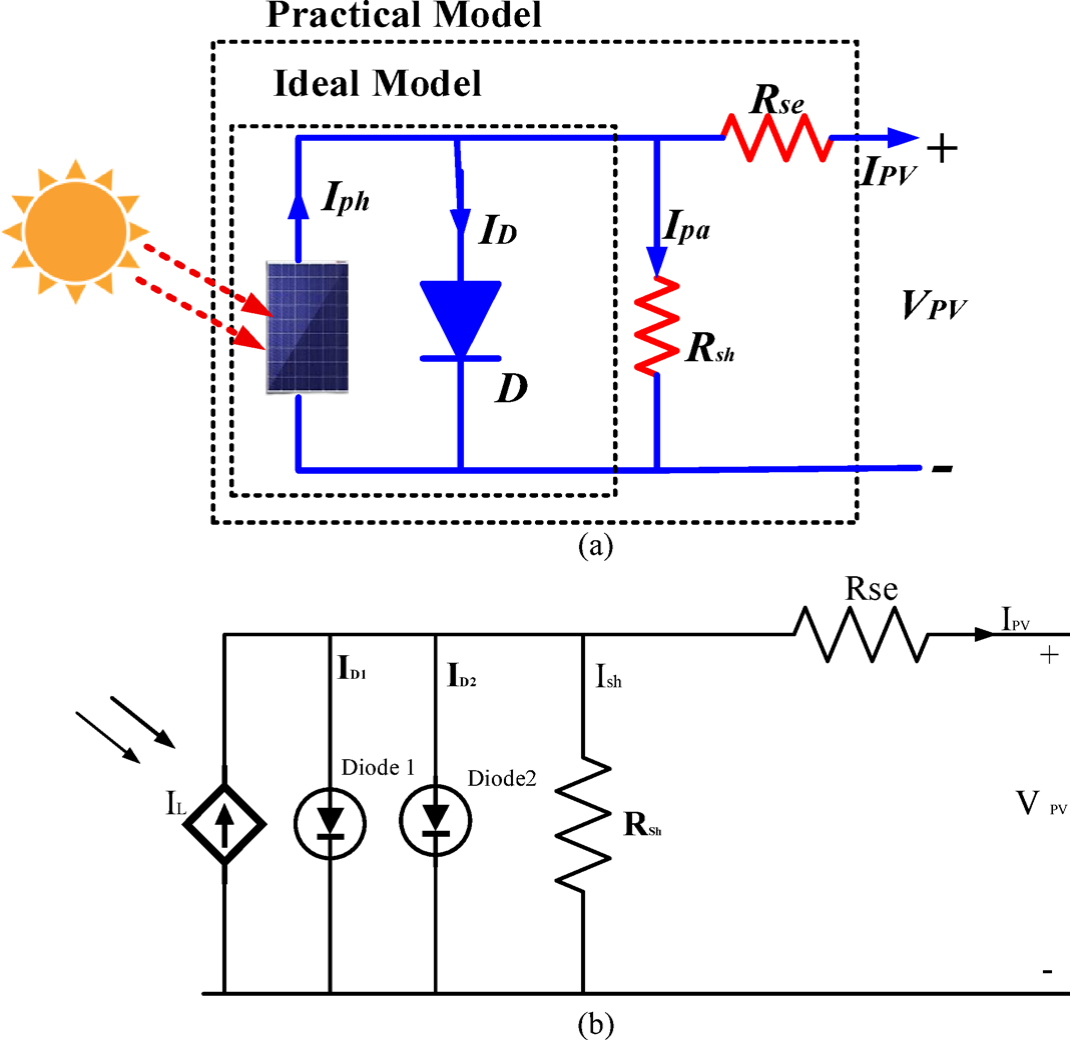
Equivalent circuits for (**a**) the one–diode model and (**b**) the double–diode model

Solar cells have a nonlinear relationship between current and voltage (I-V), so they can’t be modeled as a constant current or voltage source. Instead, their electrical behavior is represented using equivalent circuits, as shown in Fig. 2a and b. The one-diode and double-diode models are the most widely used models, providing a more accurate depiction of solar cells’ operation.

The one-diode model simulates solar cells due to its advantages over the double-diode model. It offers sufficient accuracy for fault detection and steady-state analysis at the system level, aligns with the available data for most PV modules on the market, and provides fast response times in the simulation environment^17^. Equation 1 describes the I-V characteristics of a PV panel with Ns based on the one-diode model and the properties of p-n semiconductors.

1 $$~{\text{I}}_{{{\text{PV}}}} = {\text{ I}}_{{{\text{ph}}}} - {\text{ I}}_{{\text{D}}} \left( {{\text{exp}}\left[ {\frac{{q\left( {V_{{pv}} + I_{{PV}} R_{{se}} } \right)}}{{N_{{s~}} kTa}}} \right] - {\text{1}}} \right) - \frac{{V_{{pv}} + I_{{PV}} R_{{se}} }}{{R_{{sh}} }}$$

2 $${\text{I}}_{{{\text{PV}}}} = {\text{ I}}_{{{\text{ph}}}} - {\text{ I}}_{{{\text{D}}0}} \left( {{\text{exp}}\left[ {\frac{{q\left( {V_{{pv}} + I_{{PV}} R_{{se}} } \right)}}{{N_{{s~}} k,Ta}}} \right] - 1} \right)~ - \frac{{V_{{pv}} + I_{{PV}} R_{{se}} }}{{R_{{sh}} }}$$

Where, Iph – current source, I_D0_ – leakage current of the diode, q - charge$\:,\:{N}_{s\:}$– Total cells in series, $\:k$ - Boltzmann constant, $\:T$ -solar cell’s temperature (K), $\:\text{a}$ – ideality factor, $\:{R}_{se}$ and $\:{R}_{sh}$ - Series and shunt resistance, $\:{V}_{pv}$and $\:{I}_{PV}$ -PV voltage and current.

The photocurrent is dependent on irradiance(G) and cell temperature(T)^18^ which is given by Eq. 2:

3 $${\text{I}}_{{\text{L}}} = {\text{ }}\left\{ {{\text{I}}_{{{\text{L}}0}} + {\text{ C}}_{{\text{T}}} \left( {{\text{T }}{-}{\text{ T}}_{{\text{r}}} } \right)} \right\}\frac{G}{{G_{0} }}$$

Where, I_L0_ – photocurrent at reference irradiance and temperature, C_T_ -Temperature coefficient, T_r_ -Reference temperature (25 °C), G – solar irradiance and – reference irradiance (1000 w/m2).

The reverse saturation current s changes with the solar cell surface temperature (T). It can be written as,

4 $${\text{I}}_{{\text{S}}} = {\text{ I}}_{{{\text{S}}0}} \left. {\left( {~\frac{T}{{T_{r} }}} \right.} \right)^{3} {\text{exp }}\left\{ {\frac{{qE_{g} }}{{kA}}\left[ {\frac{1}{{T_{r} }} - \frac{1}{{\text{T}}}} \right]} \right\}$$

Where, I_S0_ – reverse saturation current at reference temperature, $\:{E}_{g}$ – Energy bandgap.

The bandgap energy of a PV module depends on the semiconductor material used. At room temperature, it is approximately 1.12 eV for crystalline silicon, 1.03 eV for copper indium diselenide (CIS), 1.7 eV for amorphous silicon, and 1.5 eV for cadmium telluride (CdTe)^19^.

### Modelling algorithm

**Fig. 3:**
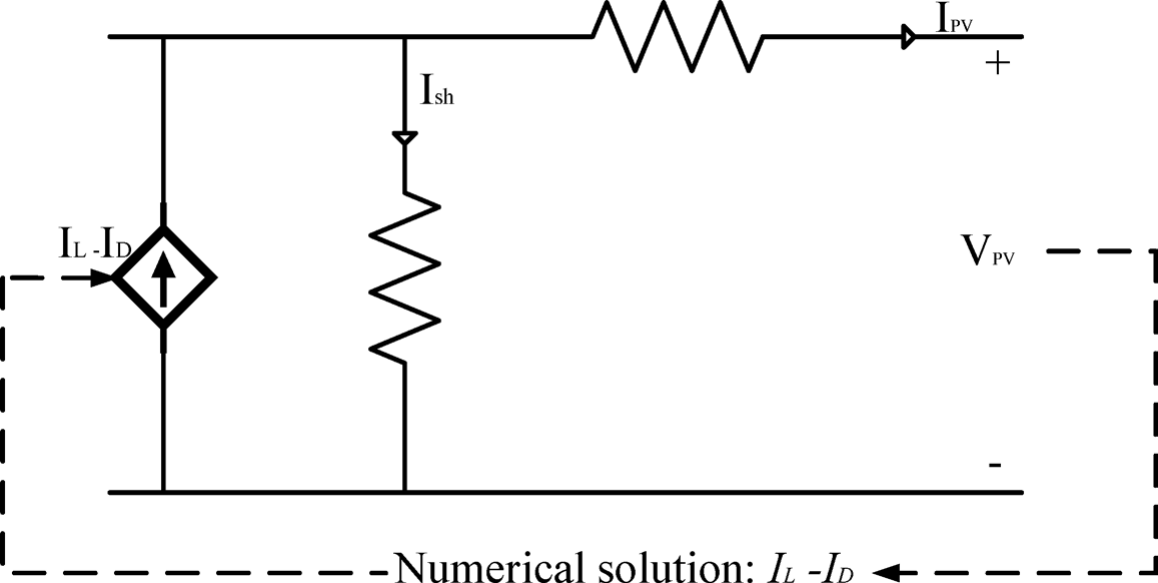
The numerical one – diode model for a PV module.

Solar cells within the same module generally receive similar irradiance levels in real-world conditions. Because of this, all the solar cells in a given PV module can be assumed to have the same characteristics and operating conditions. This allows the PV module to be treated as a basic unit consisting of identical solar cells. As a result, modeling and simulating PV modules are essential for analyzing a PV system’s regular operation and fault conditions^20^. A bypass diode is typically connected in parallel with multiple cells to improve performance under nonuniform conditions. A bypass diode is generally connected in parallel with various cells to enhance performance under nonuniform conditions. In the one-diode model of PV modules shown in Fig. 3, the modeling algorithm takes voltage (_PV_), temperature (), and irradiance () as input parameters. It then solves the relevant equations to determine the mathematical solution for current () and applies it to a controlled current source.

## Fault analysis in PV system

### OC fault

The most typical causes of these disturbances are cable junction failures, problems with overhead transmission lines, and melting of conductors or fuses in one or more phases^21^. When electrical protection devices, like fuses or circuit breakers, fail to break all three stages during a failure, one or two may open while the other remain closed. These are called series faults and can also arise when one or more conductors fail^22^.

Series failures cause an increase in voltage and frequency while decreasing current in the impacted phases. They are classified as asymmetrical or unbalanced defects. OC problems can last longer than SC faults, but should be resolved soon to avoid additional harm to the electrical system.

### SC fault

An SC happens when two sites with differing electrical potentials make an unusual connection with very low impedance. These are the most prevalent and serious problems that enable abnormal currents to pass through transmission lines or equipment. If not addressed quickly, even a short duration of these faults can cause significant damage to the equipment.

Shunt faults are another name for SC faults. These can involve a conductor or multiple conductors making contact with the ground or each other. For example, when trees or branches fall onto power lines, they can cause line-to-ground or double line-to-ground faults. Shunt faults are characterized by a current rise, voltage, and frequency drop^23^.

### Ground fault

The grounding method used in the system influences the magnitude of GF currents. In solidly or low-impedance grounded systems, GF currents are typically elevated, frequently necessitating line disconnection to clear faults. Ground overcurrent and directional overcurrent relays are commonly employed to safeguard these systems. Detecting high-impedance ground faults in multi-grounded, four-wire systems is a complex task, as the relay must differentiate between the ground fault current and the imbalanced current resulting from phase configuration and load discrepancies^24^.

In resonant-grounded systems, a variable impedance reactor is connected to the transformer neutral to balance the phase-to-ground capacitance, resulting in a high impedance path for the zero-sequence network. This approach, referred to as the Petersen coil, allows for the clearance of approximately 80% of temporary GF without tripping the breakers. High-impedance grounded systems utilize a high-impedance resistor or reactor for grounding, and their fault characteristics resemble those of resonant-grounded systems. In these systems, detecting GF demands highly sensitive relays because the fault current is minimal. Several methods are available for detection, including conventional voltage and current-based techniques and more sophisticated methods that analyze steady-state harmonic content or transient components generated by faults. However, these advanced techniques may be less effective for high-resistance faults.

### Mismatch fault

PV module anomalies, mainly the so-called mismatch problems, are twofold: temporary and permanent. The solar cell modules might get micro-cracked because of pressure in the system that may or may not be generated by hail impacts. Regardless of the damage type, they are generally caused by irregularities in the environment or during construction. The former might result from, let’s say, wind uplift, blow-offs, or dislodging due to uplift suction. Nevertheless, the latter is the after-effect of the absence of meteorological data or the use of generic values close to average. External mismatches, including those from bypass diodes, power electronic converters, and shading, are also prevalent, with shading being particularly common in practice. Additionally, the dust buildup on a PV module’s glass surface reduces its ability to transmit light, leading to a decline in power output. On average, dust accumulation causes a power reduction of approximately 6.2% after one day, 11.8% after one week, and 18.7% after one month of exposure^25^.

The impact of shading on a single cell is also affected by the cell’s characteristics, such as its shunt and series resistance, which relate to variations in reverse current. MF can lead to overheating, causing PV cells or modules to attain elevated temperatures that may result in irreparable damage. Temporary mismatch failures generally lead to energy losses of around 5–10% in Germany and Japan and between 3 and 6% in Spain. These failures adversely affect the system’s efficiency and energy output while diminishing individual PV modules’ lifespan.

## Machine learning-based fault detection in PV systems

ML offers a promising approach for identifying and predicting problems in PV panels and systems, helping to improve fault detection. PV systems can classify faults using a range of machine-learning techniques. The extent of the problem influences the choice of approach, the quantity of the information set, and the degree of interpretation needed. Several ML techniques for classifying PV system failures are covered in this Sect.^26^.

### Random forest classification

This approach utilizes multiple models to enhance predictive performance. DTs are the fundamental building blocks in a RF classifier^26^. The prediction $\:\widehat{y}$ for the input of x is expressed as follows:

4 $$\:\widehat{y}=\frac{1}{T}\sum\:_{t=1}^{T}{h}_{t}\left(x\right)$$

Where, $\:{h}_{t}\left(x\right)$ – prediction from t^th^ DT, $\:T$ – number of trees.

Utilizing a RF Classifier:

1. Data sampling: Data distribution and bootstrapping are primarily responsible for the DT algorithm’s robustness. When multiple trees are connected to the tree, it ensures that every tree is trained on different data.
2. Feature selection: Using fewer features is a way to curb the complexity of the individual computation and the correlation of models. The model correlation is reduced when this is done. With each new tree, the model becomes more diverse and thus more powerful.
3. Tree construction: Many DTs are built and combined to develop a stronger classification model. Time and time again, the data points are partitioned using the variable attributes. After that, the splits are drawn up to maximize the data gain or minimize the measure’s purity, like Gini impurity or entropy.
4. Voting: To predict, every tree must go through its data and predict. If there are many similar trees, their outputs will likely be the same.

### Support vector machine

SVMs are supervised ML algorithms primarily used for classification and regression tasks. While SVMs are most effective for binary classification, they can also be adapted to handle multi-class problems. The core idea behind SVMs is to find a multidimensional plane that best separates the classes in the training data^27^.

The model picks a hyperplane that, for each class, has the most substantial distance—called a margin—between the closest data points from that class. These critical data points, named support vectors, set the decision line. The kind of decision boundary the SVM model creates relies on the input data and the kernel function. For linear SVMs, the decision boundary manifests as a line (or hyperplane in dimensions higher than 2). Instead of non-linear SVMs, the Dataset will be transformed into a higher dimension by the kernel function. Thus, separating the classes by a linear hyperplane in that dimension could be possible.

Training an SVM refers to adjusting the hyperplane parameters so that the resulting margin gives you the maximum; simultaneously, the errors due to misclassifications are minimized. This optimization is typically approached by solving quadratic programming problems or using convex optimization. Once trained, the SVM model can classify new data points by determining where they fall relative to the decision boundary. While SVMs are most effective for binary classification, they can also be adapted to handle multi-class problems. The core idea behind SVMs is to find a multidimensional plane that best separates the classes in the training data.

Considering that the training dataset (D_1_) was split into two groups, and it can be expressed as^27^,

5 $${D}_{1}=\left\{\left({x}_{1},{y}_{1}\right),\left({x}_{2},{y}_{2}\right).....\left({x}_{n},{y}_{n}\right)\right\}x\in\:{\mathfrak{R}}^{n},y\in\:\left\{-\text{1,1}\right\}$$

where$\:{y}_{1},\:{y}_{2}\dots\:.{y}_{n}-$ labels,, $\:{x}_{1},\:{x}_{2}\dots\:{x}_{n}$ – input vector, $\:{\mathfrak{R}}^{n}$- n dimensional feature space.

The expression for the hyperplane support vector (g$\:\left(\overrightarrow{x}\right))$ is represented as follows,

6 $${\text{g}}~\left( {\vec{x}} \right) = \left( {W^{T} \vec{x} + b} \right) = 0$$

where, $\:b$- bias, W- weight vector, and $\:\overrightarrow{x}$ -feature vector.

### Decision tree classification

The core philosophy of self-organization and ML applies to all these models, including the DT. AI Renascence is not binary; it is a mix of AI and other technologies that can be applied to create faster and more efficient systems. Consequently, designing algorithms that can handle more extensive datasets can yield more reliable results than robotics and others. This automation of datasets can enable the machines to develop their logic and reasoning structure. Subsequently, these improvements are passed on to the resulting advanced applications, including robots, diagnostic systems, etc. The information gain (IG) in DT is expressed in Eq. (7)^28^

7 $$IG\left(S,A\right)=Entropy\left(S\right)-\sum\:_{v\in\:values\left(A\right)}\frac{\left|{S}_{v}\right|}{\left|S\right|}Entropy\left({S}_{v}\right)$$

Where, S-Dataset, A - Attribute, $\:{S}_{v}$ -subset for the value $\:v$.

The DT works in the following way:

1. Data splitting: The entire dataset starts as the root node of the tree. The algorithm looks for the best feature to split the data, using criteria like information gain, Gini impurity, or entropy. This chosen feature divides the data into subsets based on its different values.
2. Recursive partitioning: Each subset, or child node, is then split further using relevant attributes. This process repeats at each level, creating a tree structure where the internal nodes represent features, and the branches represent the possible values of those features.
3. Leaf node labelling: Once the tree is fully built, each leaf node is labelled with the class that most of the training data in that node. If the data at the leaf node is mixed, the node may store a distribution that shows the proportion of each class.
4. Prediction: For new data, the DT is used to predict the class by following the decisions at each node, starting from the root and moving down to a leaf node. The predicted class is the label assigned to the corresponding leaf node^28^.

### Naïve Bayes

The Naïve Bayes algorithm is a straightforward yet powerful probabilistic classification method. It is termed “naïve” because it assumes that all features are independent, meaning that the presence or absence of one feature has no impact on another^4^.

#### Naïve Bayes classification

1. Training Phase: The algorithm computes prior probabilities and conditional probabilities. The prior probability represents the likelihood of each class in the dataset, while the conditional probability, expressed as P(feature | class), indicates the probability of a specific feature occurring given a particular class.
2. Independence Assumption: When given the class label, NB makes the assumption that every feature is conditionally independent. This presumption simplifies the assessment of probability.
3. Classification Process: When classifying a new instance, the algorithm computes the posterior probability for each class. This is accomplished through multiplying the class’s prior likelihood by the product of the conditional likelihoods of the observed characteristics. The class with the greatest posterior likelihood is then allocated to the instance^29^.

Given m possible classes C = {C_1_, C_2_,…., C_m_}, and an instance $\:x$ = ($\:x$_1_, $\:x$_2_,…., $\:x$_T_) that needs classification, where $\:x$_1_, $\:x$_2_,…., $\:x$_T_ represent the instance’s attributes and T denotes the total number of attributes, the Bayes optimal classifier assigns the instance to the class with the highest corresponding probability^27^:

8 $${\text{P}}({\text{C}}_{{\text{k}}} |x_{{\text{1}}} ,_{{\text{2}}} , \ldots .,x_{T} ) = \frac{{{\text{P}}(x1,~x2, \ldots .,~x{\text{T~}}|{\text{Ck}}){\text{P}}\left( {{\text{Ck}}} \right){\text{~}}}}{{{\text{P}}\left( {x1,~x2, \ldots .,~x{\text{T~}}} \right)}}$$

Under the naive independence assumption, where each feature is considered independent of the others given the class label, if P($\:x$_1_, $\:x$_2_,…., $\:x$_T_) remains constant and identical across all classes, the conditional probability in a NB classifier can be expressed as follows:

8 $${\text{P}}({\text{C}}_{{\text{k}}} |x_{1} ,\;x_{{\text{2}}} , \ldots .,x_{T} \alpha \mathop \prod \limits_{{i = 1}}^{T} {\text{P}}(X_{{T~}} |C_{{k~}} ){\text{P}}\left( {C_{{k~}} } \right)~$$

In a Naïve Bayes classifier, P($\:x$_i_ | C_k_) represents the probability distribution of the i^th^ attribute given the selected class C_k_. To train this classifier, it is necessary to compute P($\:x$_i_ | C_k_) for all possible values of i and k. The classifier essentially solves a set of one-dimensional density estimation problems. It is commonly assumed that the attribute values within each class follow a normal distribution. The parameters of this distribution—mean and standard deviation—can be effectively estimated using the Maximum Likelihood Estimation (MLE) method. This parameterization ensures computational efficiency and simplifies implementation in distributed environments.

### XGBoost

XGBoost is a technique for ensemble learning. A methodical way to integrate the prediction ability of several learners is through ensemble learning. A single model that provides the combined output of many models is the end result. To enhance the model’s performance, XGBoost sequentially integrates DTs, which serve as its base learners. Boosting is the technique by which each new tree is trained to fix the mistakes produced by the one before it.It can swiftly train models on big datasets because to its integrated parallel processing. Additionally, XGBoost allows for modifications, enabling users to modify model parameters to maximize performance according to the particular situation^28^.

At iteration t, we must minimize the following objective function (L),

10 $$L=\sum\:_{i=1}^{n}l\left(y-\widehat{y}\right)+\sum\:_{k=1}^{K}\varOmega\:({f}_{k})$$

Where, $\text{l}\left(\text{y}-\widehat{\text{y}}\right)$ – loss function, ${\Omega\:}\left({\text{f}}_{\text{k}}\right)$ -regularization, K – number of trees.

### Performance metrics

To evaluate classification models, commonly used metrics include accuracy (%), recall (%), precision (%), and F1 score (%).

- Accuracy (%) measures the proportion of correctly classified observations out of the total dataset.
- Precision (%) represents the fraction of correctly predicted positive instances relative to all predicted positive instances.
- Recall (%) indicates the proportion of correctly identified positive cases compared to the actual number of positive instances in the class. The classification performance metrices are expressed as follows^29^:

11 $$Accuracy = \frac{\text{T}\text{P}+\text{T}\text{N}\:}{\text{T}\text{P}+\text{T}\text{N}+\text{F}\text{P}+\text{F}\text{N}\:}$$

12 $$Recall = \frac{\text{T}\text{P}}{\text{T}\text{P}+\text{F}\text{N}\:}$$

13 $$Precision = \frac{\text{T}\text{P}}{\text{T}\text{P}+\text{F}\text{P}}$$

14 $${\text{F}}_{{\text{1}}} {\text{Score}} = {\text{2}} \times \frac{{{\text{Precision}} \times {\text{Recall~}}}}{{{\text{Precision~}} + {\text{~Recall}}}}$$

In the above equations, a True Positive (TP) occurs when a positive instance is correctly identified as positive, while a False Positive (FP) happens when a negative instance is mistakenly classified as positive. Similarly, a True Negative (TN) refers to a correctly identified negative instance, whereas a False Negative (FN) occurs when a positive instance is incorrectly labelled as negative.

### Loss function

In ML, loss functions are used to measure how accurately a model’s predictions match the actual outcomes. The equation typically involves summation symbols (Σ), which indicate that the formula is calculating the sum of the loss across all the data points in a dataset^26^. Th loss function can be expressed as follows,

15 $$\:L=-\frac{1}{\text{N}}{\sum\:}_{i=1}^{N}{\sum\:}_{j=1}^{M}{y}_{ij}\text{l}\text{o}\text{g}\left({y}_{ij}\right)$$

N-Data points, M-classes, $\:{y}_{ij}$ -true label indicator.

### Classification score

The classification score is typically understood as the model’s accuracy, indicating the percentage of correct predictions among the total input samples. It works well when there’s a balanced distribution of samples across each class in multiclass classification tasks.

### Confusion matrix

The confusion matrix is a key tool for assessing a model’s accuracy, consisting of four main components:

- True positive: Both the predicted and actual values are 1.
- True negative: Both the predicted and actual values are 0.
- False positive: The predicted value is 1, but the actual value is 0.
- False negative: The predicted value is 0, but the actual value is 1.

In binary classification, the diagonal of the matrix should have the highest values, as they represent correctly classified samples. In multi-class classification, each class corresponds to a row and column in the matrix^30^.

### Model accuracy and loss curve

Model gain and loss curves, also referred to as learning curves, are commonly used for models that learn gradually over time. These curves display both training progress and model performance, helping to illustrate how well the model has been trained overall. The loss curve shows a decreasing number of loss points and the best curve shows that the training and recognition losses gradually decrease until they become stable with less variance. Similarly, the correct and stable curve (with the smallest deviation from the true value) for training and validation indicates the best model. Regularization, dropout layers, and early stopping are some of the strategies that are used to address the issues like overfitting - the places where more focus is on the model’s performance on the training set rather than its ability to predict unseen data^31^.

Using a variety of key metrics to measure our model’s performance. The loss function defines the divergence between the predictions made by the model on the input examples and the actual outputs and tries to minimize this difference. Accuracy tells how many of the predictions are actually correct, effectively measuring overall performance. The confusion matrix is a chart that sorts predictions into true positives, false positives, and true negatives, offering a deeper look at the model’s errors. At last, learning curves illustrate the accuracy and loss trends across all training epochs by showing the progress of the model and thus guaranteeing it performs well on new data. Training the individual dataset category gives an accuracy of 97.20%. This is a good start, but it is not enough to be considered accurate.

## Result and discussion

**Fig. 4:**
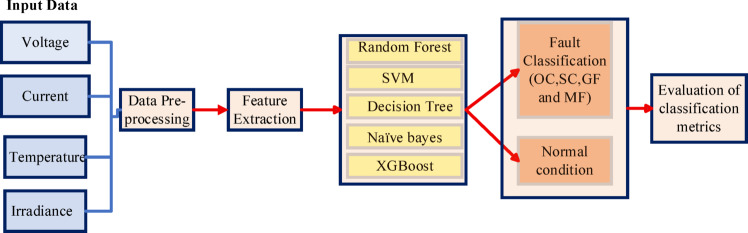
Block diagram of solar PV fault detection and classification.

The block diagram of PV fault identification and Classification is shown in Fig. 4. Various ML Algorithms like RF, SVM, DT, NB and XGBoost are proposed in this work for fault identification and classification.

*Data collection*

The initial step entails gathering data from the solar PV system. This may include sensor data like current, voltage, temperature, and light intensity.

*Preprocessing*

After data collection, the next phase is preprocessing the data. This may involve cleaning the dataset, normalizing values, addressing missing data, and labeling, among other tasks.

*Model training*

A ML model is developed using the pre-processed data. 80% of the data set is reserved for training purposes. During this stage, the model learns to recognize various types of faults by detecting patterns in the data provided.

*Fault classification*

Once the model has been trained, it is capable of classifying incoming data. The model examines the new input and provides a classification result that indicates the type of defect, if any, that is present.

*Evaluation*

The model’s efficacy is assessed utilizing a range of metrics. Using a confusion matrix, we can derive accuracy, precision, recall, and F1 score.

**Table 1:** Benchmarking ranges of parameters for various fault Types.

Sl.No	Fault type	Power min	Power max	Voltage Min	Voltage max	Current min	Current max	Temp min	Temp max
1	GF	64.96	95.76	84.12	111.94	0.69	1.44	25	34
2	MF	75.83	104.68	351	410.98	0.17	0.32	25	34
3	Normal	83.79	119.26	373.03	426.32	0.2	0.3	25	34
4	OC	0	0	370.6	431.93	0	0	25	34
5	SC	0	0	0	0	0.75	1.41	25	34

The PV’s real-world data was gathered under a range of operational circumstances. Table 1 displays the benchmarking parameter ranges for the different types of faults. These ranges are used to analyse the PV system’s performance as benchmarking reference values. Particularly, MF and GF only exhibit a partial drop in power production, but open and SC faults are distinguished by the total failure of power generation. Strong characteristics for precise categorization are provided by the diverse voltage and current ranges, which also help to distinguish between various types of faults.

**Table 2:** Key dataset and simulation parameters.

Sl.No.	Parameter	Description
1	Dataset size	20,000 samples
2	Sampling frequency	1 ms (1000 Hz)
3	Simulation duration	2 s per run
4	Fault introduction time	Typically at 0.5 s into simulation
5	Irradiance range	600 W/m² to 1000 W/m²
6	Ambient temperature range	25–40 °C
7	Noise handling	Gaussian noise with 1% std. deviation of signal
8	Noise filtering	Simple moving average smoothing
9	Training/testing splits	80% training and 20% testing
10	Feature scaling	Standardization

The dataset used for training and evaluation was derived from MATLAB-based simulations of a 9 × 9 PV array under varied fault conditions. The data set and other simulation parameter details are shown in Table 2. Each simulation recorded key electrical parameters at a fixed time step of 1 ms, over a total simulation duration of 2 s. This resulted in approximately 20,000 samples per simulation scenario, capturing the dynamic transition of electrical behaviour post-fault introduction (typically at 0.5 s).To reflect environmental variability, the simulation scenarios incorporated typical irradiance levels ranging from 600 W/m² to 1000 W/m² and ambient temperatures from 25 °C to 40 °C. The PV module behaviour was modelled accordingly to reflect the impact of these variations on fault manifestation. Gaussian noise with a standard deviation of 1% of the signal magnitude was applied to the voltage and current readings to assess model robustness under real-world conditions. Prior to training, this noise was filtered using a simple moving average smoothing method to reduce sharp transient anomalies while retaining fault-relevant transitions.

SC, OC, GF, and MF are detected based on variations in input parameters such as voltage, current, power, and temperature.

*SC* This scenario is characterized by a sudden decrease in voltage to almost zero volts and a sudden increase in current, often much higher than usual. It usually denotes a low-resistance, direct transmission that avoids the load.

*OC* In this case, the voltage stays near its open-circuit value (about 500 V), but the current drops to 0 A. That is a clear sign that the circuit has broken or disconnected.

*GF* Here, we observed an unanticipated current to ground or a decrease in insulation resistance, while the voltage stayed relatively constant. This indicates that unintentional grounding is causing the current to leak.

*MF* These are identified when, even in the presence of equal sunshine, there is a decrease (often 20–30%) in power or current from one or more panels. Shade, dirt, age, or module flaws are the leading causes.

**Fig. 5:**
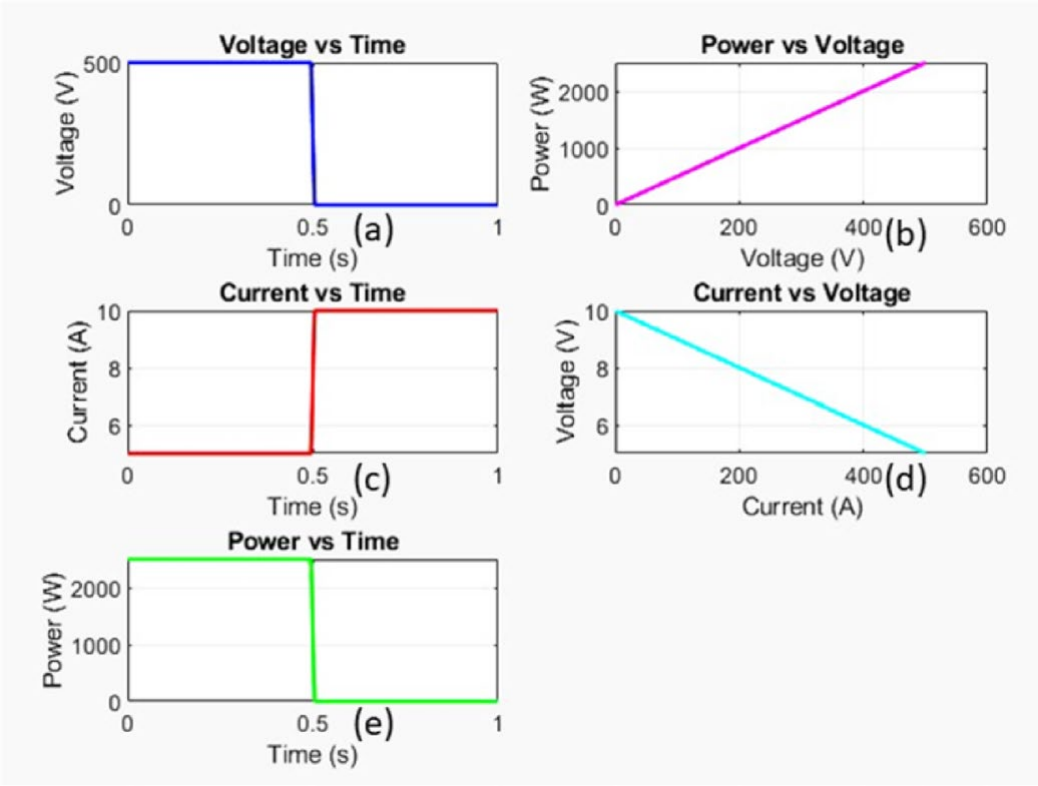
Simulation response of SC fault.

Figure 5 represents the MATLAB simulation of the SC fault and the various curves.

- Voltage versus time
  It shows that the voltage remains constant at around 500 V until approximately 0.5s, when it drops sharply to 0 V.

b) Power versus voltage
  Depicts a linear increase in power as voltage increases, suggesting a direct proportional relationship.

c) Current versus time
  Current stays constant at around 6 A until 0.5s, then jumps to 10 A.

d) Current versus voltage
  Shows a negative slope, indicating an inverse relationship between voltage and current.

e) Power versus time
  Power remains steady at around 2000 W and suddenly drops to zero at 0.5s.

**Fig. 6:**
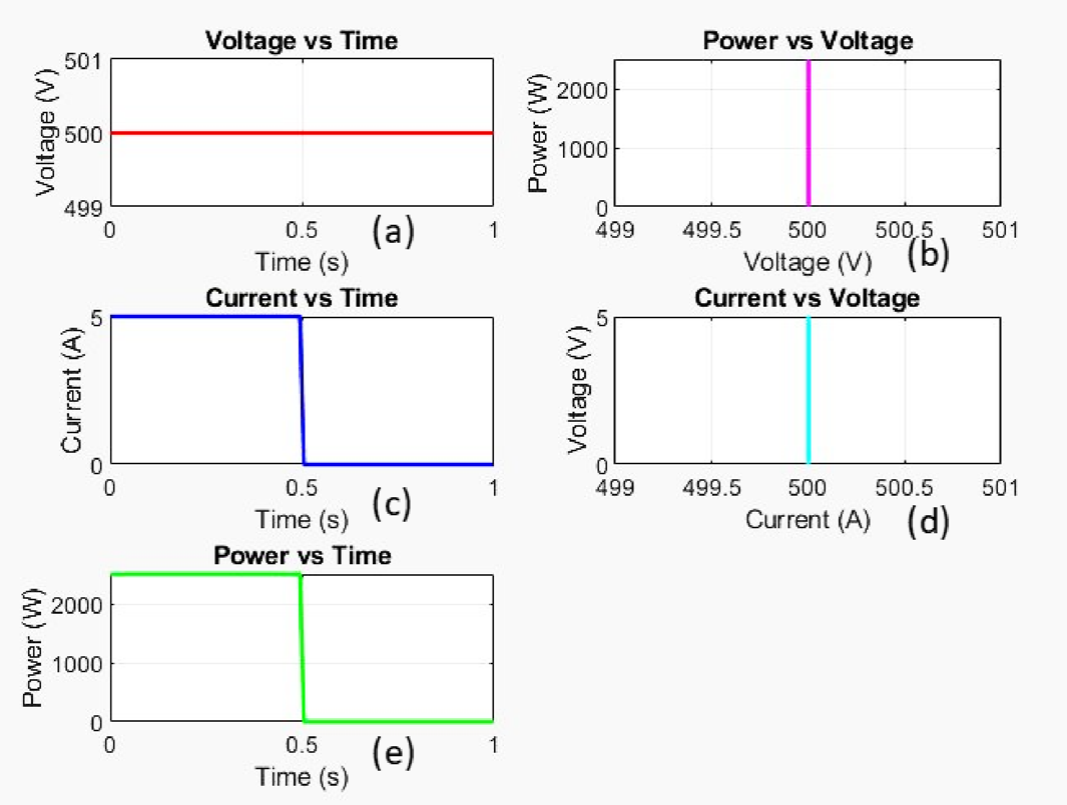
Simulation response of OC fault.

Figure 6 represent the MATLAB simulation response of the OC fault.

- Voltage versus time
  The voltage remains constant at 500 V throughout the observed period, with no noticeable change.

b) Power versus voltage
  The power vs. voltage relationship is represented as a vertical line at 500 V, implying that power is only present at this voltage level.

c) Current versus time
  The current remains steady at 5 A until around 0.5s, after which it suddenly drops to 0 A.

d) Current versus voltage
  The plot shows a vertical line at 500 V, indicating that the voltage remained constant at 500 V, independent of the current.

e) Power versus time
  The power is constant at 2500 W (500 V × 5 A) until 0.5s, then drops to 0 W.

**Fig. 7:**
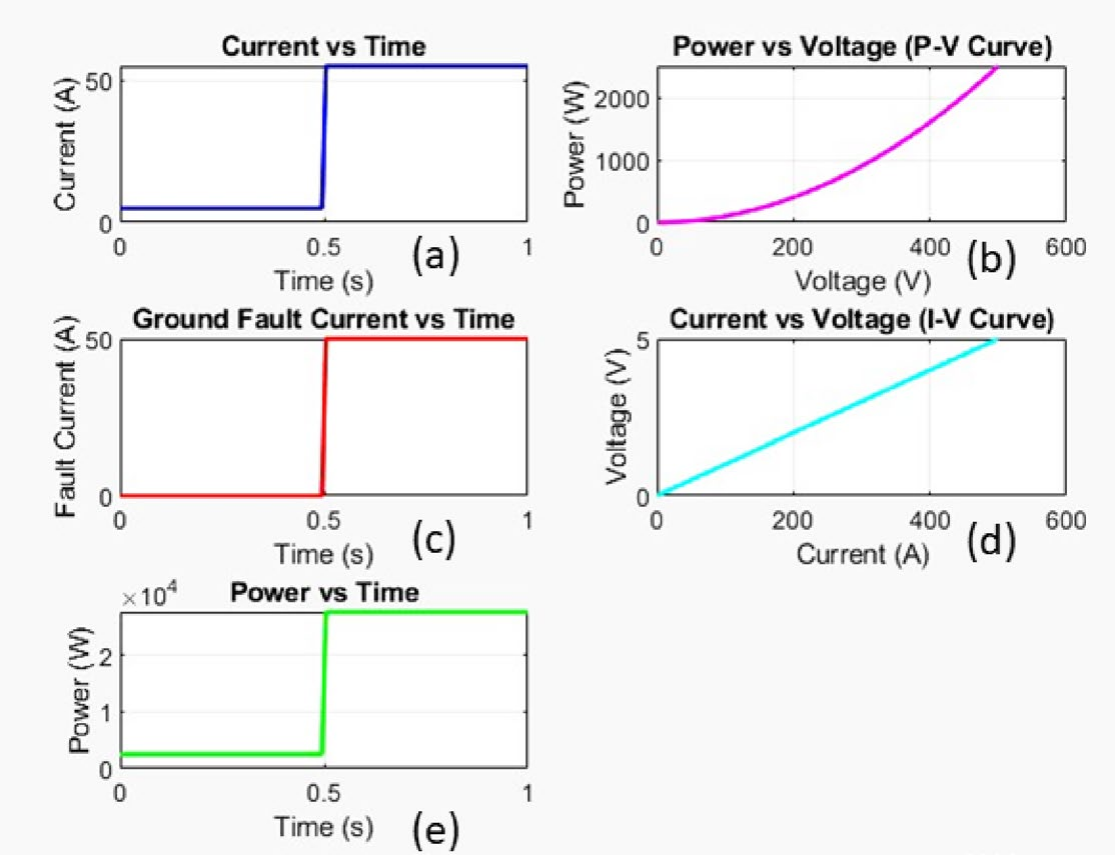
Simulation response of ground fault.

Figure 7 represent the MATLAB simulation response of the ground fault.

- Current versus time
  Initially, the current is at 0 A but jumps sharply to around 50 A at 0.5s, indicating a sudden load activation or system change.

b) Power versus voltage
  Shows a parabolic relationship, meaning power increases non-linearly as voltage increases.
c) Ground fault current versus time
  The fault current is zero initially but jumps to around 50 A at 0.5s, indicating a ground fault occurring at that moment.

d) Current versus voltage
  Displays a linear relationship, meaning the voltage increases proportionally with current.

e) Power versus time
  Power is initially low but suddenly increases to around 20,000 W (2 × 10⁴ W) at 0.5s, showing a significant power surge.

**Fig. 8:**
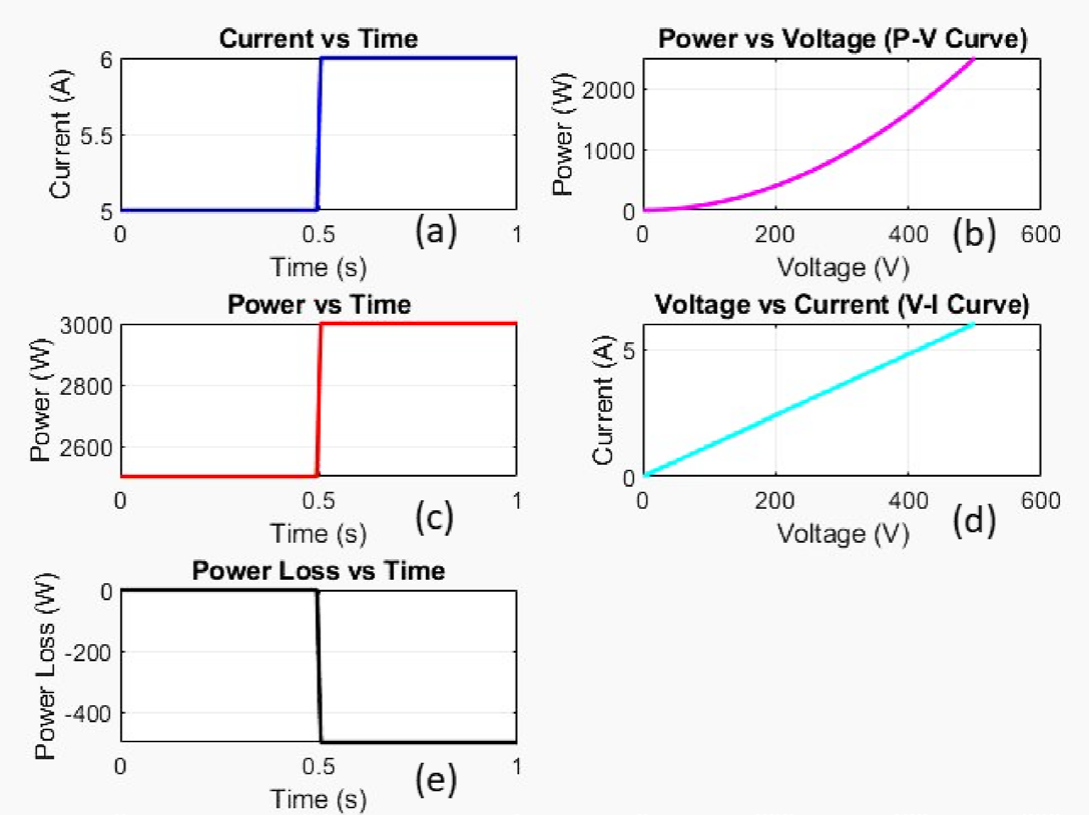
Simulation response of mismatch fault.

Figure 8 represent the MATLAB simulation response of the mismatch fault.

- Current versus time
  The current starts at 5 A and suddenly jumps to about 6 A at 0.5s.
  This suggests a load increase or system event at t = 0.5s.

b) Power versus voltage
  The power increases non-linearly with voltage.

c) Power versus time
  The power remains constant initially (around 2600 W) and jumps to 3000 W at 0.5s.

d) Voltage versus current
  Displays a linear relationship between voltage and current.

e) Power loss versus time
  The power loss is zero before 0.5s, but suddenly becomes negative after 0.5s.

**Fig. 9:**
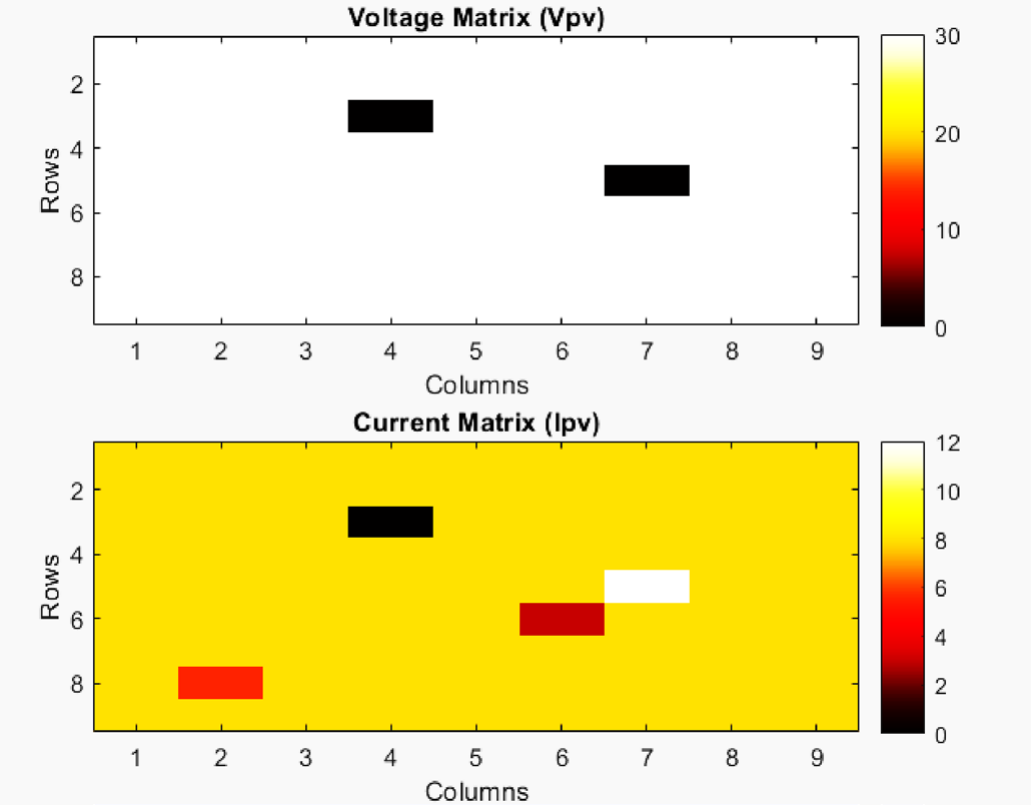
Voltage matrix and current matrix.

**Fig. 10:**
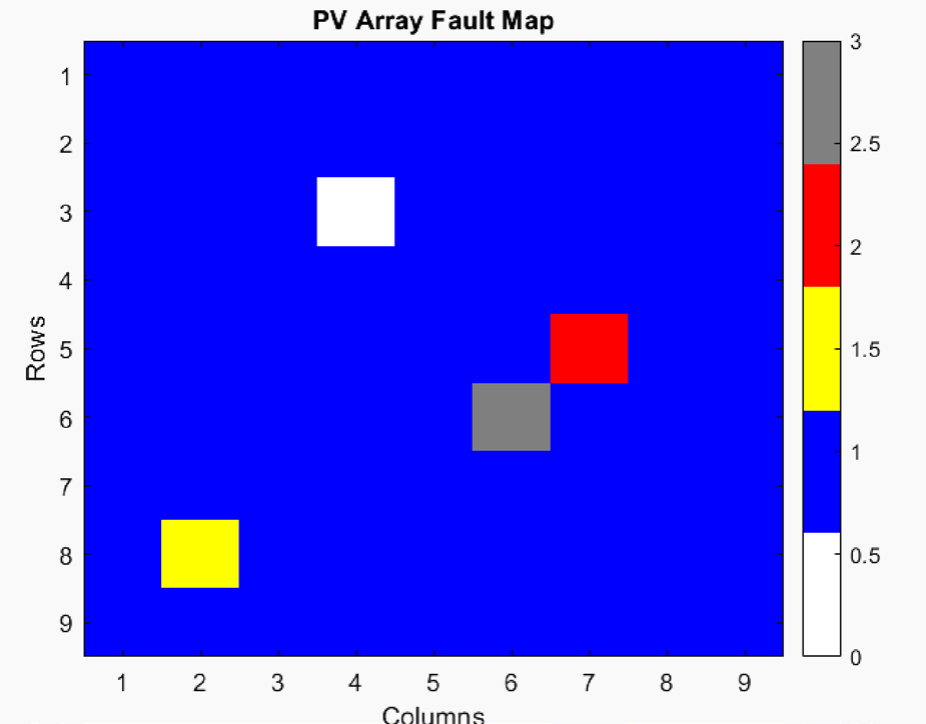
PV Fault array map.

The Voltage Matrix and Current Matrix are represented in Fig. 9. The 9 × 9 PV array fault analysis successfully simulated and identified different fault conditions within the PV system. The PV Fault array map is shown in Fig. 10. The results from the simulation provide insights into how various faults impact the PV module’s electrical characteristics.

1. Normal operation
  Modules operating under normal conditions maintain a voltage of 30 V and a current of 8 A per module.

2. OC fault (at position (3,4))
  Voltage drops to 0 V and current to 0 A, indicating complete isolation from power generation.

3. Mismatch fault (at position (8,2))
  Voltage remains unchanged at 30 V, but the current is reduced to 5.6 A (70% of the nominal value) due to mismatched characteristics.

4. SC fault (at position (5,7))
  Voltage drops to 0 V, while the current rises to 12 A (150% of nominal value), increasing the risk of module damage.

5. Ground fault (at position (6,6))
  Voltage remains at 30 V, but the current is limited to 3 A, determined by the ground fault resistance (10Ω).

Overall system output:

- Total voltage output (V_total_): The summed voltage across rows varies depending on fault locations.
- Total current output (I_total_): The summed current across columns is impacted by the mismatch and short-circuit faults.
- Total power output (P_total_): Approximately 18.4 kW, significantly lower than the expected power in a fault-free scenario, indicating efficiency loss due to faults.

**Fig. 11:**
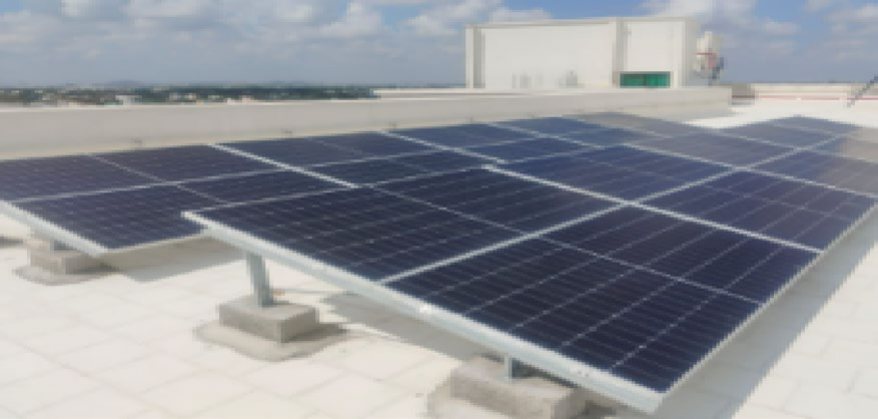
Setup of 10 MW PV panel.

**Fig. 12:**
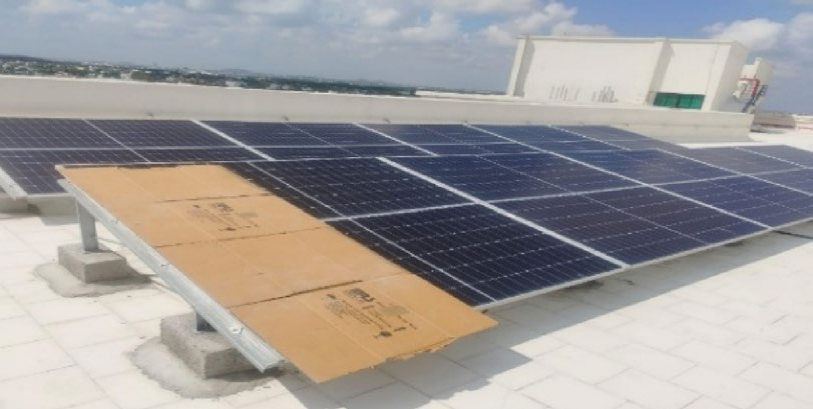
Setup of 10 MW shaded PV panel.

The setup of the 10 MW PV panel is shown in Fig. 11. We hide a part of the Solar PV panel to get the shading effect, also known as mismatch fault, as shown in Fig. 12.

Voltage, current, temperature, irradiance, and other electrical and environmental data have been collected through modeling and real-world measurements. A 9 × 9 PV array was modeled in the simulation environment using MATLAB/Simulink. Using native measuring instruments, voltage and current were measured under various operating situations, including typical and fault scenarios such as OC, SC, GF, and MF.

The simulation’s temperature and irradiance levels were manually adjusted to replicate real-world environmental variables, including shade. Calibrated sensors connected to a data-collecting system were used to manage voltage and current during the tests. A pyranometer positioned at a tilt angle like the PV panels was used to monitor irradiance. In contrast, thermocouples and digital temperature sensors were used to detect the panel surface and surrounding air temperatures. To guarantee that the data remained accurate, all of the measurement apparatus was checked often. I-V and P-V characteristics were then created using the gathered data in a one-diode PV model. These values are used as input for supervised learning configurations of machine learning models after being labeled and arranged into datasets.

**Fig. 13:**
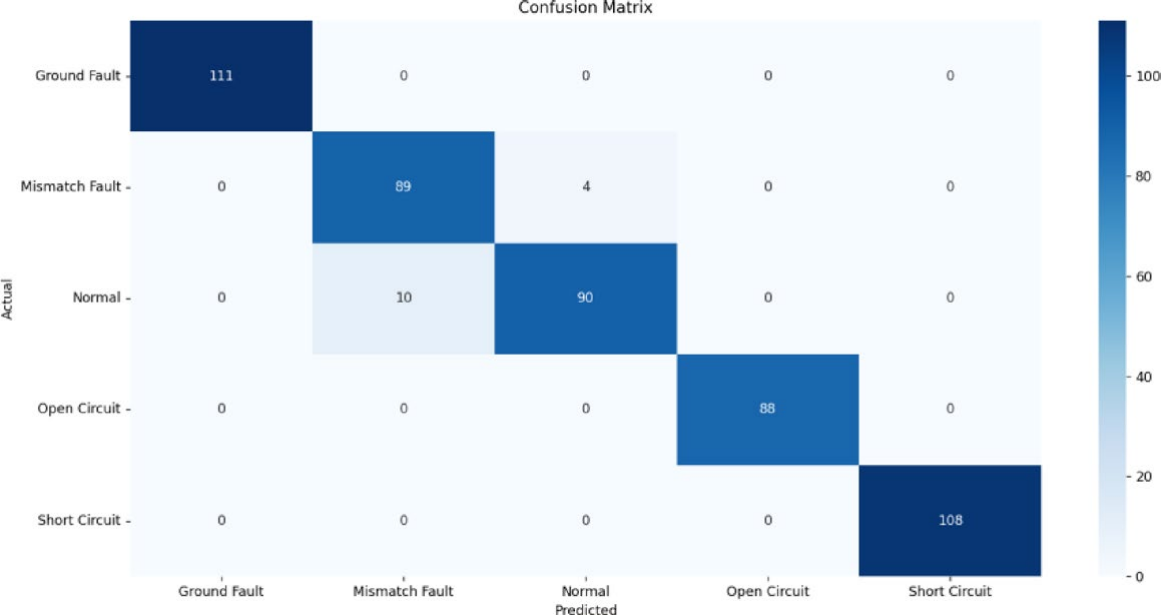
Confusion matrix of RF classification.

**Fig. 14:**
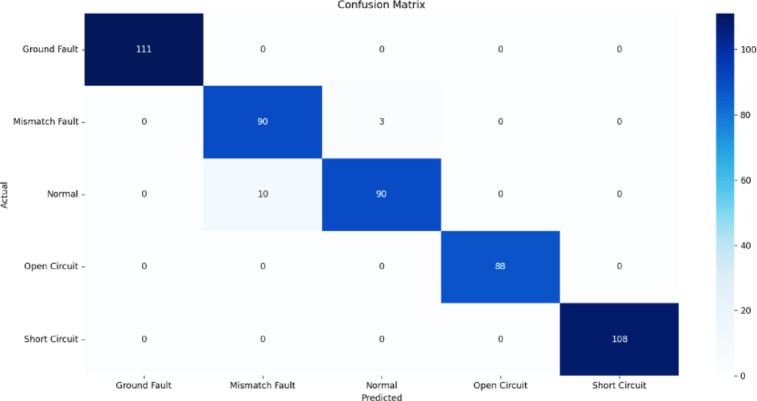
Confusion matrix of SVM.

**Fig. 15:**
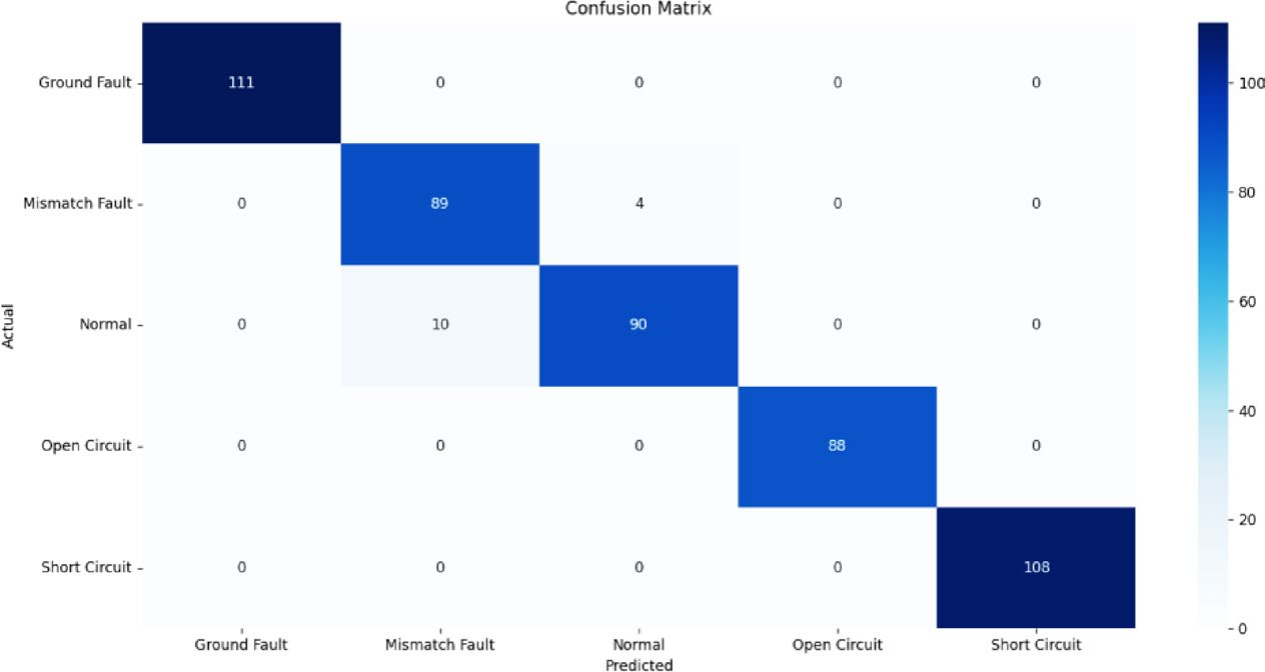
Confusion matrix of DT.

**Fig. 16:**
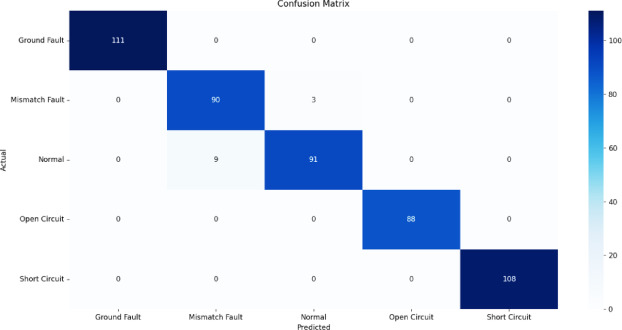
Confusion matrix of Naïve Bayes.

**Fig. 17:**
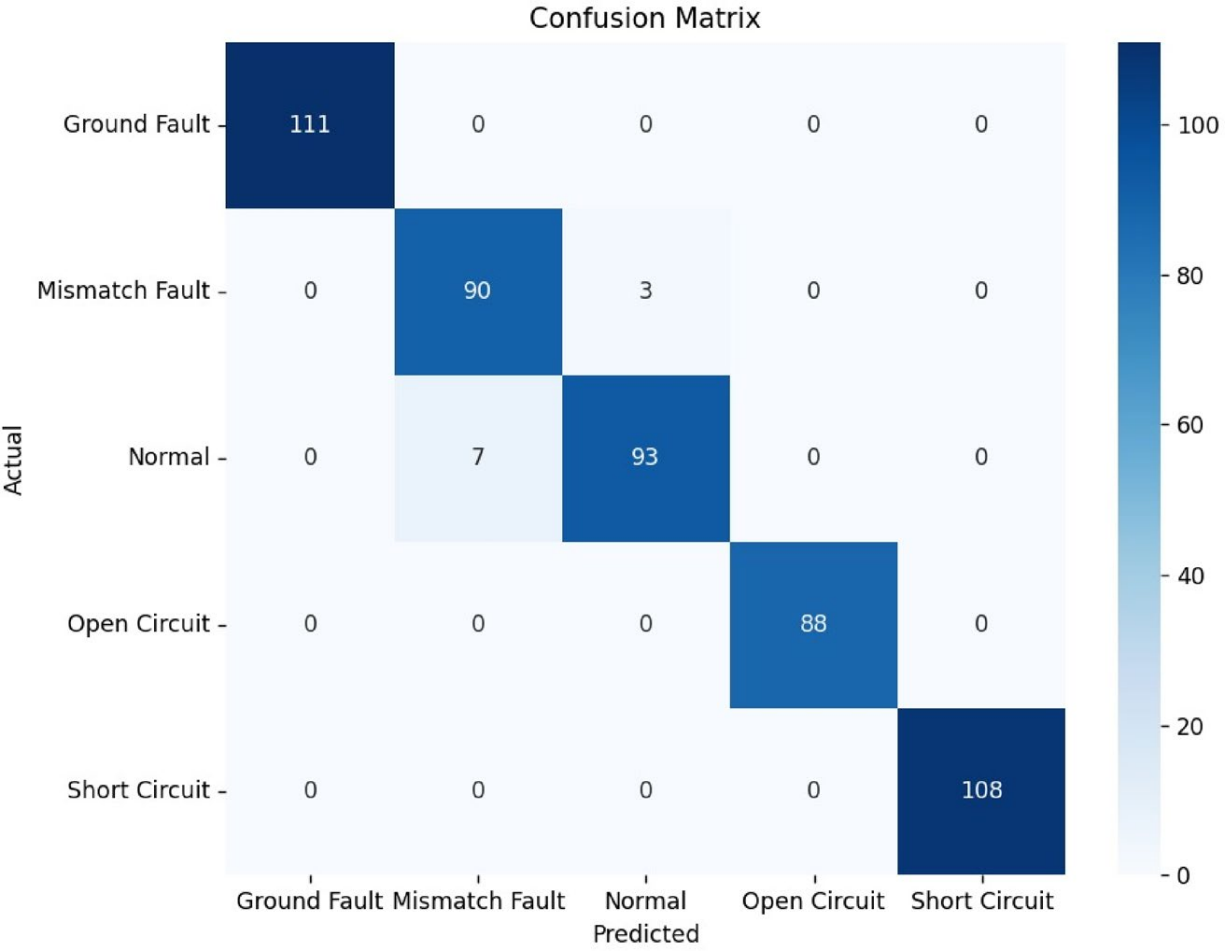
Confusion matrix of XGBoost.

Figures 13, 14, 15, 16, 17 illustrate the confusion matrices corresponding to the performance of the RF Classifier, SVM, DT, NB, and XGBoost algorithms. The confusion matrix reveals the model successfully distinguishes between different fault types, with highly accurate favorable rates across all categories. Misclassifications occurred in a few instances where fault conditions exhibited similar electrical characteristics, leading to minor overlaps in prediction. The above figures visualize the confusion matrix, confirming the classifier’s effectiveness in differentiating faults.

**Fig. 18:**
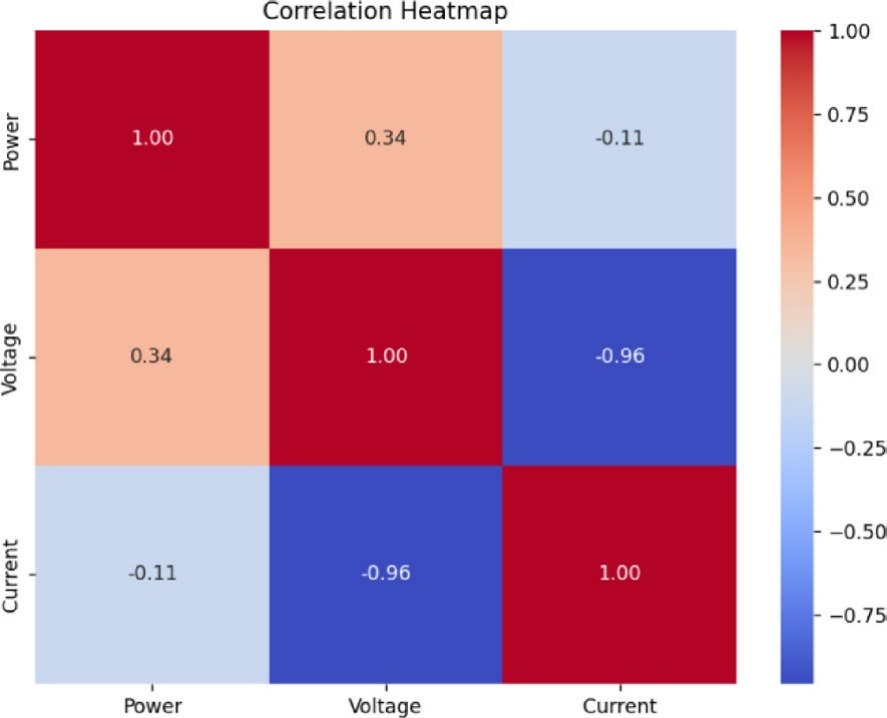
Correlation heatmap of RF classification.

**Fig. 19:**
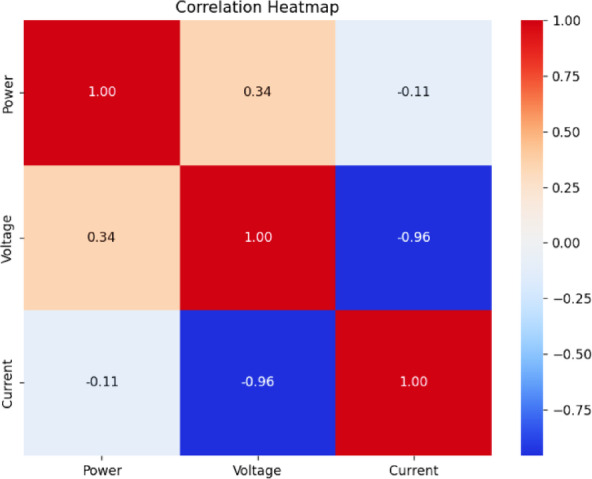
Correlation heatmap of SVM.

**Fig. 20:**
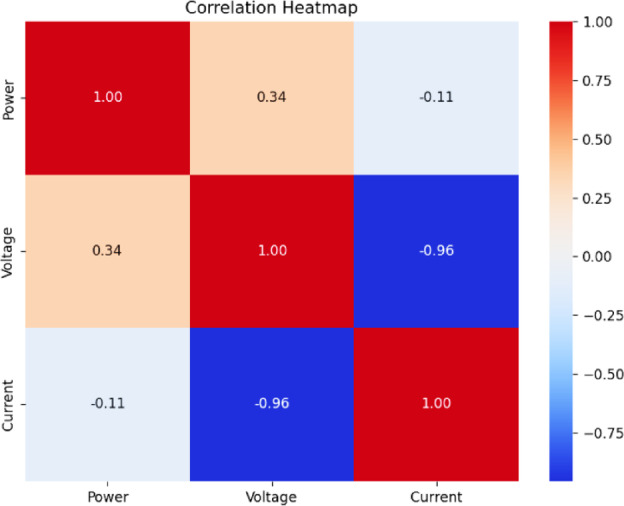
Correlation heatmap of DT.

**Fig. 21:**
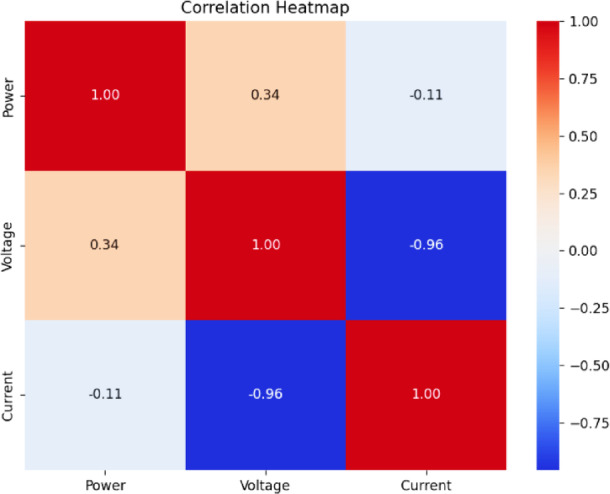
Correlation heatmap of Naïve Bayes.

**Fig. 22:**
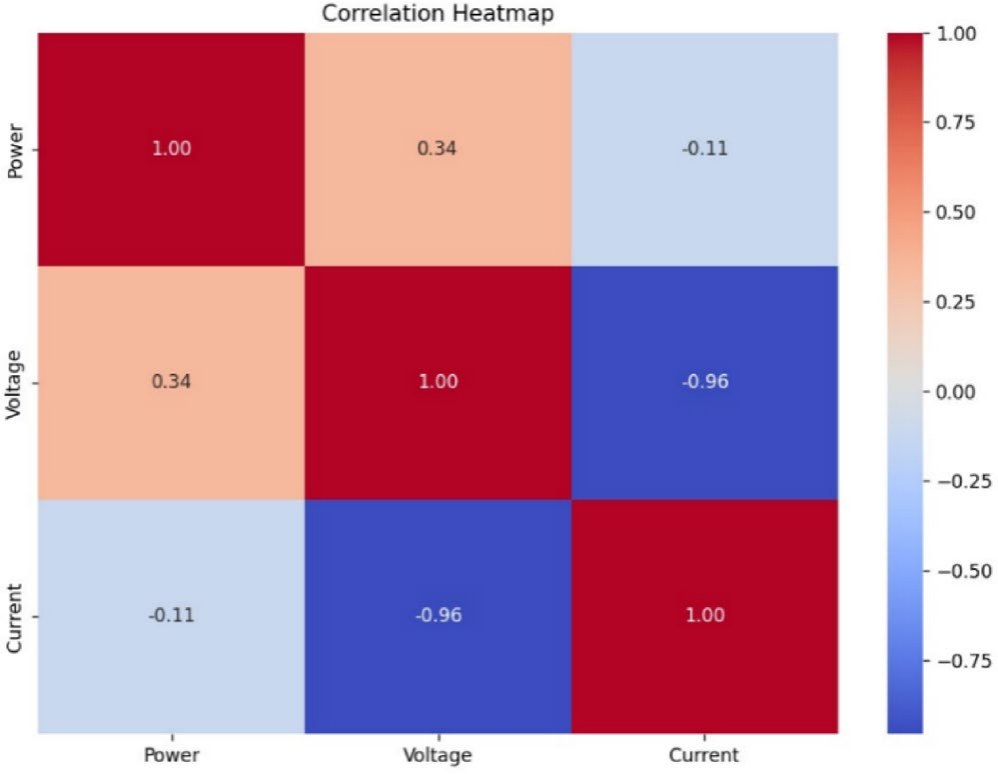
Correlation heatmap of XGBoost.

Figures 21, 22 depict the correlation heatmaps for the RF Classifier, SVM, DT, NB, and XGBoost algorithms. A correlation heatmap (highlights the relationships between electrical parameters—power, voltage, and current. The analysis indicates strong correlations between power and voltage, suggesting that certain fault conditions significantly affect these variables. This information helps refine feature selection and make the model easier to interpret.

**Fig. 23:**
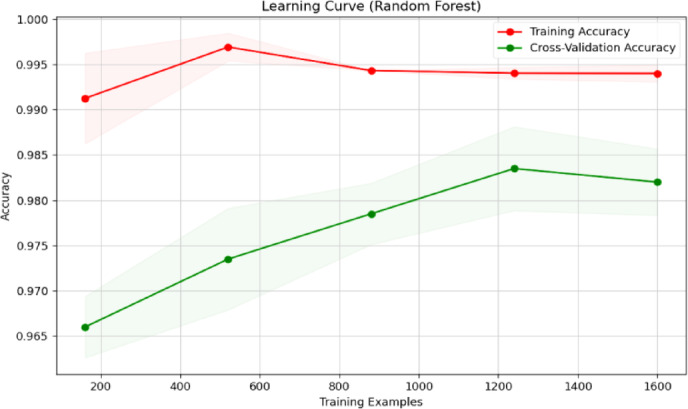
Learning curve of RF classification.

**Fig. 24:**
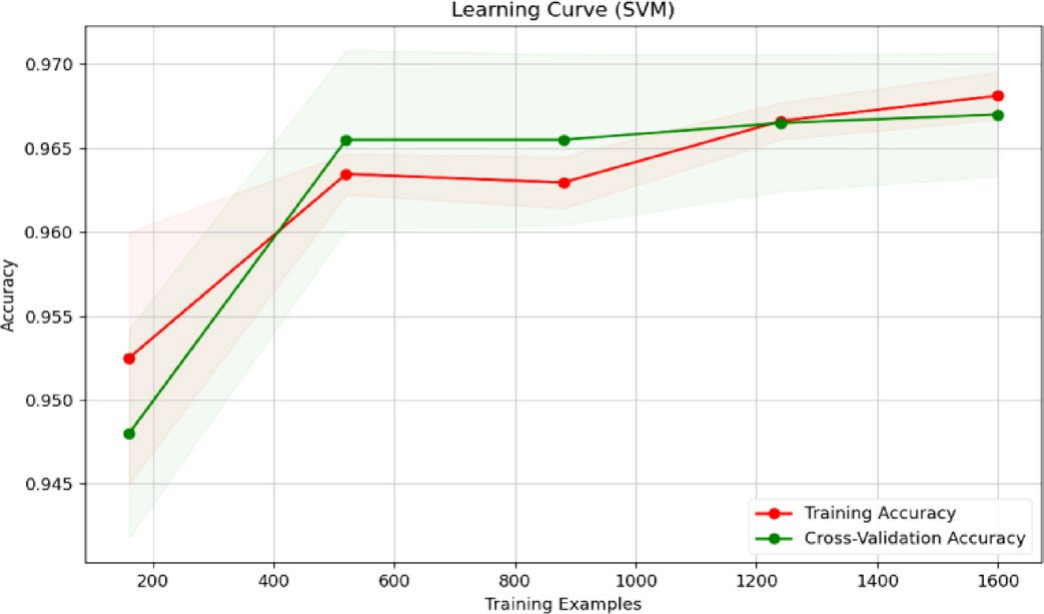
Learning curve of SVM.

**Fig. 25:**
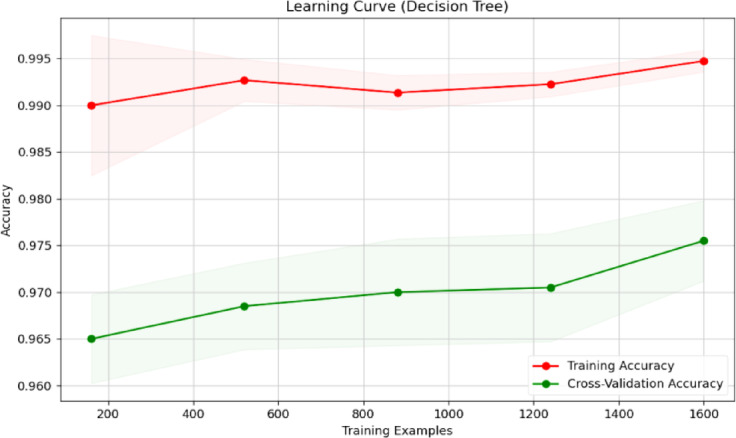
Learning curve of DT.

**Fig. 26:**
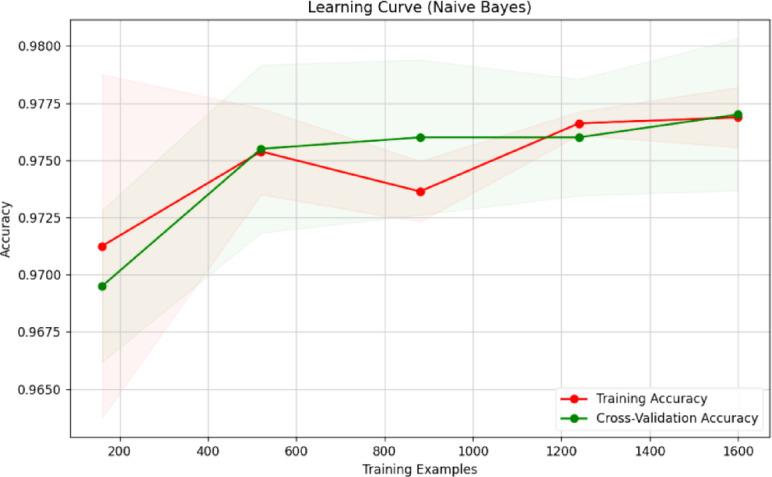
Learning curve of NB.

**Fig. 27:**
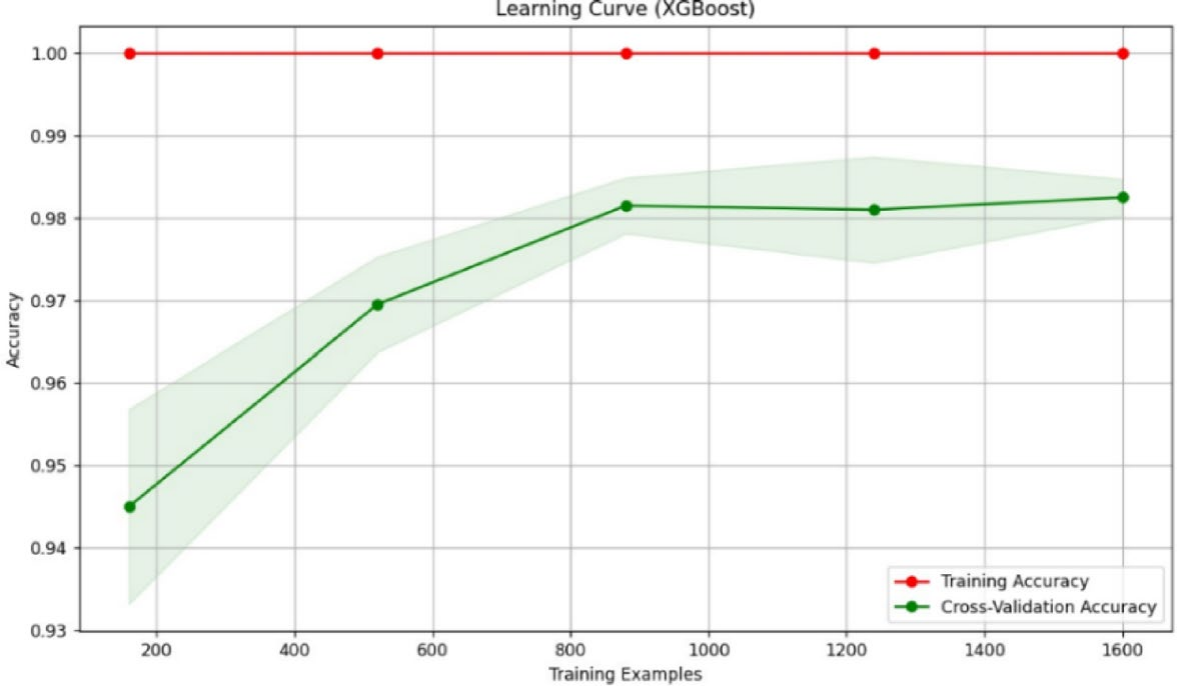
Learning curve of XGBoost.

Figures 23, 24, 25, 26, 27 depict the learning curve of the RF Classifier, SVM, DT, NB, and XGBoost algorithms. The learning curve illustrates the model’s performance over increasing training dataset sizes. The results show that the training accuracy remains stable, while the validation accuracy converges at a certain number once it’s evaluated, indicating that the model generalizes well without overfitting. The consistent gap between training and validation performance suggests room for further optimization, possibly through additional feature engineering or hyperparameter tuning.

### Performance from interpretation

**Table 3:** Comparison of various ML algorithm under fault Cases.

ML Algorithms	Classification	Precision	Recall	F1-score	Accuracy (%)
RF	GF	1	1	1	97.20
	MF	0.90	0.96	0.93	
	Normal	0.96	0.90	0.93	
	OC	1	1	1	
	SC	1	1	1	
SVM	GF	1	1	1	97.40
	MF	0.90	0.97	0.93	
	Normal	0.97	0.90	0.93	
	OC	1	1	1	
	SC	1	1	1	
DT	GF	1	1	1	97.20
	MF	0.90	0.96	0.93	
	Normal	0.96	0.90	0.93	
	OC	1	1	1	
	SC	1	1	1	
Naïve Bayes	GF	1	1	1	97.60
	MF	0.91	0.97	0.94	
	Normal	0.97	0.91	0.94	
	OC	1	1	1	
	SC	1	1	1	
XGBoost	GF	1	1	1	98.00
	MF	0.93	0.97	0.95	
	Normal	0.97	0.93	0.95	
	OC	1	1	1	
	SC	1	1	1	

The comparison of various ML algorithms under fault cases is mentioned in Table 3. Comparing the performance of several ML methods shows that all models have good classification accuracy, ranging from 97.20 to 98%. With a maximum accuracy of 98% and better precision and recall in identifying Mismatch Faults and Normal situations, the XGBoost performed better than the others.

**Table 4:** Comparison of model training time and complexity.

Model	Training Time (s)	Complexity	Suitable for Real-Time
NB	~ 0.3	Low (O(n))	Yes
DT	~ 1.2	Medium (O (n log n))	Moderate
RF	~ 3.8	High (O(ntrees·n))	Not ideal
SVM	~ 2.5–4.0	High (O(n^2^))	Not ideal
XGBoost	~ 3.5	High(O (k d n log n))	Moderate

*k- Boosting rounds, n -training samples, d-maximum depth of each DT.

The comparison of model training time and complexity is shown in Table 4. According to the results, the Gaussian NB model trained in less than a second, using three essential features like power, voltage, and current. It reduces training time by enabling closed-form probability calculation based on the condition of feature independence. On the other hand, because of ensemble structures and kernel changes, models such as SVM and RF require more computing power. We also tested XGBoost, which showed better handling of non-linear feature interactions and a slightly higher accuracy, even though it needed a more extended training period (~ 3.5s). XGBoost’s effective regularization and scalability make it a good fit for cloud-based systems with additional resources. NB and XGBoost have complementary benefits that allow them to be implemented in various solar PV monitoring settings based on accuracy and latency.

## Discussion

A comparative evaluation of the amount of research on PV system fault assessment, fault categories taken into account, ML techniques, accuracy, outcomes and limitaions is shown in Table 5. While references cover a variety of fault types, few take mismatch issues into account. ML methods such as RF, SVM, DT, NB, and XGBoost have been used selectively throughout the experiments. The proposed work, on the other hand, sets itself apart by incorporating a comprehensive strategy that addresses all significant PV issues and uses several ML methods to improve system dependability and diagnostic accuracy.

**Table 5:** Comparison of proposed work with existing works.

Reference number	Fault assessment	Types of Faults				Machine learning algorithm					Accuracy	Outcomes	limitations
		OC	SC	GF	MF	RF	SVM	DT	NB	XGBoost			
^4^		✔				✔	✔	✔	✔		99%	Fault detection in healthy and faulty panels	Fault detection is computed only in OC faults
^11^				✔	✔						100%	Fault detection using thermography and NN	Overfitting issues
^13^				✔							98%	Analysis of GF detection methods	Lacks on-site experimentation
^15^					✔						Not Reported	passive and active techniques for mismatch fault detection techniques	need for advanced control algorithms
^14^		✔	✔								Not Reported	Analysis of model-based fault detection techniques	Requires time-consuming tuning for each PV installation
^26^							✔				97.2%	Hilbert-Huang Transform and ML based fault detction	Fault detection is not computed other than SVM
^27^		✔	✔			✔	✔	✔	✔		99%	ML based Fault detection	Fault detection is computed only in OC and SC faults
Proposed work	✔	✔	✔	✔	✔	✔	✔	✔	✔	✔	98%	Fault assessment and ML based fault detection	Fault detection is computed for fixed system configuration

## Conclusion

The findings from the MATLAB simulations offer essential insights into how PV systems behave under various fault scenarios, including SC, OC, MF, and GF. These faults significantly affect system efficiency by changing voltage, current, and power characteristics. The findings suggest that one method of fault, open-circuit faults, may allow no power, but only a decrease in efficiency may be brought about by other kinds of faults. The fault detection system driven by ML support succeeds in using classifiers such as RF, SVM, DT, NB and XGBoost. XGBoost attained high classification accuracy of 98% and thus are considered a real method for detecting and resolving system inefficiencies.

To discover hotspot-related problems early, the proposed work was expanded to incorporate Convolutional Neural Networks (CNNs) for thermographic picture analysis of PV modules. Using the sequential nature of power and voltage data, recurrent neural networks (RNNs), particularly LSTMs, will be investigated for time-series-based defect prediction. A cloud-connected IoT-based alerting system will also be developed to provide real-time defect notification to distant maintenance units. Also, Future research will concentrate on confirming the findings using actual PV system data from publicly accessible sources like TIGO Energy and PVOutput.org to improve the reliability and generality of the proposed fault detection algorithms.

Additional research can investigate incorporating battery storage to reduce energy loss caused by PV faults, thereby maintaining a steady energy supply. Studies aimed at refining PV system designs for enhanced fault tolerance and implementing predictive maintenance techniques will improve system dependability. Tackling these issues in future research can significantly advance the creation of more efficient, intelligent, and resilient PV systems, thereby promoting renewable energy and sustainability.

## Data Availability

The datasets used and/or analysed during the current study available from the corresponding author on reasonable request.

## Abbreviations

PV: Photo voltaic
AI: Artificial intelligence
DL: Deep learning
ML: Machine learning
IV: Current–voltage
PV: Power–voltage
IoT: Internet of Things
MW: Mega watt
SVM: Support vector machine
CIS: copper indium diselenide
CdTe: cadmium telluride
OC: Open circuit
SC: Short circuit
MF: Mismatch Fault
GF: Ground fault
NB: Naïve Bayes
DT: Decision tree
RF: Random forest
